# Rotavirus Infection: A Systemic Illness?

**DOI:** 10.1371/journal.pmed.0040117

**Published:** 2007-04-17

**Authors:** David C. A Candy

## Abstract

Candy discusses a new study in*PLoS Medicine* that challenges the view that in children with rotavirus diarrhea, the virus is confined to the upper small intestine.

Since the 1950s, rotavirus has been recognized in veterinary circles as an important cause of diarrhoea in young livestock and poultry. In the 1970s, the virus was found to be a cause of infantile diarrhoea in humans [1], and after this discovery rotavirus rapidly became established as the most prevalent cause of paediatric diarrhoea [2].

## Pathology of Rotavirus Infection

Rotavirus preferentially infects the mature villous enterocytes (intestinal epithelial cells) of the upper small intestine [3]. The microcirculation of jejunal villi responds (by constricting and dilating) to infection in infant mice, but viral particles are not seen by electron microscopy in the tissues of the lamina propria beneath the epithelial layer or within mucosal blood vessels [3]. Inflammatory changes in the jejunal epithelium and lamina propria are not striking compared with those seen with invasive bacterial enteropathogens such as Salmonellae and Shigellae.

Many infants with rotavirus are febrile and have symptoms of upper respiratory infection. This presentation was ascribed by clinicians to coincidental infection with respiratory viruses, endemic in the rotavirus season. There have been sporadic reports that suggested that rotavirus may infect organs other than the gut, such as the nervous system [4], but the ultra-structural changes found in the gut have led to the conclusion that rotavirus infection is confined to the mucosa of the upper small intestine.

## A New Study Showing Rotavirus Antigenaemia

A new study published in *PLoS Medicine* challenges the view that the virus is confined to the upper small intestine in children with rotavirus diarrhoea [5]. In the study, Blutt and colleagues found rotaviral antigens and RNA in blood samples from children with rotavirus diarrhoea [5].

The researchers tested serum samples obtained upon hospitalisation from children with gastroenteritis (57 stool rotavirus-positive and 41 rotavirus-negative), children with diagnosed bronchiolitis of known (*n* = 58) or unknown (*n* = 17) viral aetiology, children with non-infectious, non-chronic conditions (*n* = 17), and healthy adults (*n* = 28). They confirmed that 90% of children with rotaviral diarrhoea have rotavirus antigenaemia compared with 12% with rotavirus-negative diarrhoea, 12% with virus-negative bronchiolitis, and 0% in children and adults without diarrhoea. The children with rotavirus antigenaemia and rotavirus-negative diarrhoea may have had undetected rotavirus infection; this was proven serologically in half of the children for whom paired sera were available. All children with rotavirus in the stool had infectious viruses in their plasma.

The strength of this study was that infective, transmissible rotavirus was found in blood samples from humans in a large series of patients. The researchers did not simply rely on animal data or the detection of viral fragments such as viral-associated proteins or RNA.

## Implications of the Study

The authors conclude that the finding of infectious rotavirus in the blood suggests extra-intestinal involvement in rotavirus pathogenesis, though they concede that the impact of rotavirus viraemia on clinical manifestations of infection is unknown. One possibility—which needs further investigation—is that the extra-intestinal manifestations of rotavirus infection, such as respiratory symptoms and seizures, are in fact due to the infection being systemic rather than localised to the jejunal mucosa.

This study also confirms the previous suggestion that rotavirus diarrhoea can be diagnosed by assay of serum, as well as stool antigen [6]. The detection of current rotavirus infections in epidemiological studies will no longer rely on obtaining stool samples.

Prevention of rotavirus by immunisation has recently been achieved with an attenuated live rotavirus. Live, attenuated poliovirus is being replaced in the United Kingdom by the more convenient and safer killed virus. Now that rotavirus has been shown to be a systemic infection, it will be interesting to see whether the same fate will befall the live rotavirus vaccine. It would be informative to test this hypothesis in animal models.

## 

**Figure:**
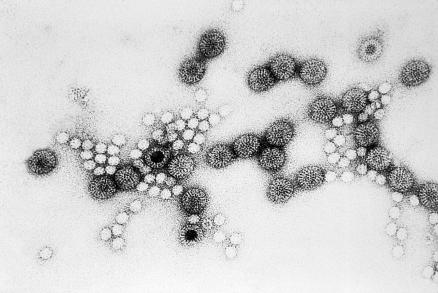
Electron micrograph showing a number of RNA rotavirus virions (which have a wheel-like appearance) and a number of unknown, 29 nm virion particles

## References

[pmed-0040117-b001] Bishop RF, Davidson GP, Holmes IH, Ruck BJ (1973). Virus particles in epithelial cells of duodenal mucosa from children with acute non-bacterial gastroenteritis. Lancet.

[pmed-0040117-b002] Shornikova AV, Casas IA, Isolauri E, Mykkanen H, Vesikari T (1997). Lactobacillus reuteri as a therapeutic agent in acute diarrhea in young children. J Pediatr Gastroenterol Nutr.

[pmed-0040117-b003] Osborne MP, Haddon SJ, Spencer AJ, Collins J, Starkey WG (1988). An electron microscopic investigation of time-related changes in the intestine of neonatal mice infected with murine rotavirus. J Pediatr Gastroenterol Nutr.

[pmed-0040117-b004] Lynch M, Lee B, Azimi P, Gentsch J, Glaser C (2001). Rotavirus and central nervous system symptoms: Cause or contaminant? Case reports and review. Clin Infect Dis.

[pmed-0040117-b005] Blutt SE, Matson DO, Crawford SE, Staat MA, Azimi P (2007). Rotavirus antigenemia in children is associated with viremia. PLoS Med.

[pmed-0040117-b006] Blutt SE, Kirkwood CD, Parreno V, Warfield KL, Ciarlet M (2003). Rotavirus antigenaemia and viraemia: A common event?. Lancet.

